# A codon-shuffling method to prevent reversion during production of replication-defective herpesvirus stocks: Implications for herpesvirus vaccines

**DOI:** 10.1038/srep44404

**Published:** 2017-03-13

**Authors:** Gang Li, Charles Ward, Rukhsana Yeasmin, Steven Skiena, Laurie T. Krug, J. Craig Forrest

**Affiliations:** 1 Department of Microbiology and Immunology and Center for Microbial Pathogenesis and Host Inflammatory Responses, University of Arkansas for Medical Sciences, Little Rock, Arkansas, USA; 2 Department of Computer Science, Stony Brook University, Stony Brook,New York, USA; 3 Department of Molecular Genetics and Microbiology, Stony Brook University, Stony Brook, New York, USA

## Abstract

Herpesviruses establish life-long chronic infections that place infected hosts at risk for severe disease. Herpesvirus genomes readily undergo homologous recombination (HR) during productive replication, often leading to wild-type (WT) reversion during complementation of replication-defective and attenuated viruses via HR with the helper gene provided *in trans*. To overcome this barrier, we developed a synthetic-biology approach based on a technique known as codon shuffling. Computer-assisted algorithms redistribute codons in a helper gene, thereby eliminating regions of homology, while enabling manipulation of factors such as codon-pair bias and CpG content to effectively titrate helper-gene protein levels. We apply this technique to rescue the replication of a murine gammaherpesvirus engineered with a mutation in the major immediate-early transactivator protein RTA. Complementation with codon-shuffled RTA constructs did not yield any WT revertant virus, a sharp contrast to WT virus contamination frequently observed during complementation with an unmodified helper gene. We further demonstrate the importance of eliminating WT virus contamination in an animal model of gammaherpesvirus lethality. We propose complementation by codon shuffling as a means to produce replication-defective or attenuated viruses. This method has immediate utility for investigating roles of essential genes in viral replication and will better enable future development of herpesvirus vaccines.

The *Herpesviridae* family of viruses (herpesviruses) includes large, enveloped viruses with a double-strand DNA genome^1^. Herpesviruses are ubiquitous, and infections can cause morbidity and mortality in livestock, wildlife, and the human population. The nine known human herpesviruses cause a wide range of diseases. Mild infection outcomes include childhood rashes caused by the roseoloviruses (human herpesviruses (HHV)-6A, HHV-6B, and HHV-7) or fever blisters and genital lesions due to herpes simplex viruses (HSV-1 and HSV-2), among others^1^. However, human herpesviruses also cause congenital birth defects (human cytomegalovirus, HCMV) and potentially lethal diseases such as encephalitis (HSV-1 or HSV-2), fulminant hepatitis (HCMV), and numerous cancers caused by Epstein-Barr virus (EBV) and Kaposi sarcoma-associated herpesvirus (KSHV)^1^.

The herpesvirus infectious cycle is characterized by two distinct phases^1^. After primary exposure an acute, productive phase of infection known as the lytic cycle ensues during which viral progeny are produced and tissue damage can occur due to viral replication and the host response to infection. Once primary infection is controlled by the host immune response, herpesviruses enter a chronic phase of infection known as latency in which viral genomes are maintained with minimal gene expression in target cells, and viral clearance by the immune system is avoided. Latent herpesviruses retain the capacity to re-enter the productive phase, a process known as reactivation. Once acquired, a herpesvirus infection is incurable, as these viruses establish chronic infections that persist for the life of the infected host. Infected persons therefore remain at risk for developing severe manifestations from herpesvirus infections throughout their lifetimes, especially individuals whose immune systems are compromised by HIV infection, immunosuppressive drugs, or age. Preventive or prophylactic vaccines are only available for varicella-zoster virus, an attenuated virus derived by serial passage in cell culture that protects against chicken pox, a manifestation of primary infection, and shingles, a result of reactivation from latency^2^.

Herpesviruses (and other DNA viruses) readily undergo homologous recombination during their productive replication cycles, a property harnessed for decades in the generation of recombinant viruses. However, the recombinogenic nature of the viral genome poses a major problem in efforts to generate replication-defective viruses and potential vaccine strains. This is because a replication-defective or severely attenuated mutant virus must be complemented for replication through the use of a helper gene provided *in trans* in a producer cell line. Homologous recombination between the mutant viral genome and the helper gene often yields contamination of the mutant virus stock with wild-type (WT), replication-competent virus. Work with HSV mutants suggests that large deletions of complete genes within the viral genome might alleviate this complication^3^. However, this approach is not feasible when large deletions lead to polar effects on the expression of other genes, such as overlapping and convergent ORFs and noncoding RNAs^4^,^5^. In addition, retention of a partial gene sequence might be required to maintain antigenic epitopes. Therefore, methods are needed prevent the problem of homologous recombination in settings where discreet mutations in a viral gene are warranted.

Employing synthetic biology, it is possible to create helper genes with completely novel sequences and reduced homology to a viral genome. Codon shuffling is a process whereby codon usage frequency and amino acid sequence is preserved for a given coding sequence, but with synonymous codons shuffled like a deck of cards using computer-assisted algorithms^6^,^7^,^8^. Importantly, this type of synthetic manipulation to alter codon distribution in a viral gene effectively changes the nucleotide sequence in a gene of interest without altering the amino acid sequence. In addition, the genetic deck can be manipulated to change any number of variables to potentially increase or decrease protein levels, a consideration for viral gene products that are toxic to the host cell^9^. Previously used to attenuate live viruses such as poliovirus and influenza virus^6^,^7^,^8^, we repurposed the codon-shuffling algorithm to design synthetic complementation constructs in which regions of nucleotide homology to a viral genome are minimized to prevent recombination, yet factors that favor protein production of the complementing construct are maintained or enhanced. Experiments described here demonstrate that codon shuffling provides a method for generating high-titer stocks of a replication-incompetent herpesvirus that is completely devoid of WT virus contamination. We further demonstrate *in vivo* the importance of using such an approach to prevent reversion in a potential vaccine stock.

## Results and Discussion

### Test system and codon shuffled construct design

We sought to determine whether codon shuffling of helper genes provides an effective method for complementing mutant herpesvirus. As a test system, we chose murine gammaherpesvirus 68 (MHV68) – a well-characterized rodent gammaherpesvirus that is genetically related to human pathogens EBV and KSHV and exhibits parallel strategies for persistence in the host^10^,^11^,^12^. The replication and transcription activator protein, RTA, is an immediate-early viral gene product encoded by open reading from 50 (*ORF50*) that is absolutely essential for initiation of the gene-expression cascade that drives lytic viral replication^13^. The requirement for RTA in MHV68 replication was demonstrated using a recombinant virus in which a translation stop codon and frameshift mutation were inserted into the RTA coding sequence of *ORF50* (ORF50.Stop, RTA-null MHV68) at amino acid 116 in RTA protein^13^. Stocks of the RTA-null virus can only be produced in helper cells engineered to express WT RTA protein^13^. Given the propensity of herpesviruses to undergo homologous recombination and the necessity for RTA in replication, it is not surprising that WT reversion occurs frequently during the generation of RTA-null virus stocks in complementing cell lines (1 WT revertant in 1 × 10^5^–3 × 10^4^ PFU)^13^. Hence, RTA-null virus provides an ideal and stringent test of the utility of the codon-shuffling approach for complementation.

We designed five unique codon-shuffled (CS) RTA complementation constructs, designated CS-RTA1-5, using a codon-shuffling algorithm as detailed in the Materials and Methods^6^. Alignments of CS-RTA constructs to WT RTA-encoding nucleotide sequence are shown in Supplementary Figure 1. To briefly summarize, all synthetic RTA constructs maintain the same frequency of codon usage as WT RTA (referred to as the codon-adaptation index, (Table 1), but differ with regard to percent identity to WT sequence and the degree of change in codon-pair bias (CPB) and CpG content – factors potentially influenced by codon shuffling that can influence translation efficiency^9^ (Table 1). CS-RTA1 and CS-RTA2 have the most nucleotide changes across the entire ORF, but CS-RTA2 has a more optimal CPB score than CS-RTA1. Since the mutation that generates a stop codon in the ORF50.stop mutant is in the 5′ region of the ORF, we reasoned that reversion-associated homologous recombination might be limited to the genomic region directly 5′ and 3′ to the stop mutation. CS-RTA3 is a fusion of the first 381 nt of CS-RTA1 with the remainder of the WT RTA sequence, resulting in 136 nt changes and a negative CPB score. CS-RTA4 restores a more optimal CPB score, and was only applied to the first 381 nt of *ORF50*, resulting in 109 nt changes. CS-RTA5 is a fusion of the first 384 nt of CS-RTA2 with the remainder of the WT RTA sequence, leading to 133 nt changes and a corresponding optimal CPB.

### Validating functionality of codon-shuffled helper genes

**Figure 1:**
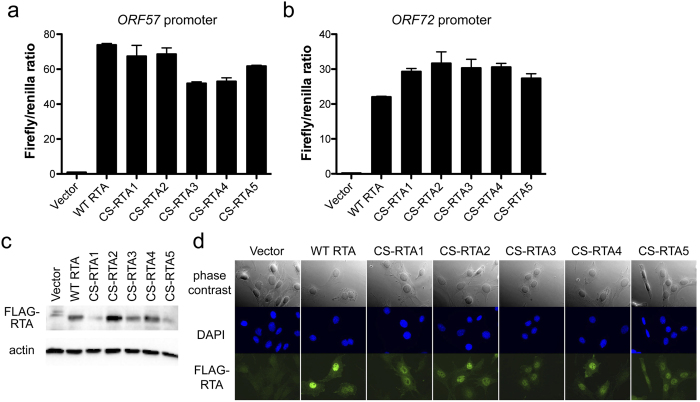
CS-RTA constructs are translated and functional. (**a** and **b**) 293 T cells were transfected with plasmids encoding the indicated constructs in the presence of firefly luciferase reporter plasmids containing RTA-responsive promoter sequences for *ORF57* (**a**) or *ORF72* (**b**). Cells were harvested 24 h post-transfection, and luciferase activity in lysates was determined in a luminometer. Experiments were normalized for variation in transfection efficiency by co-tranfection with a constitutively-active renilla luciferase reporter plasmid. Values indicate RTA-mediated induction of the viral promoters as a firefly/renilla signal ratio. Values are averages from three independent experiments. Error bars represent standard deviations. (**c** and **d**) NIH 3T12 fibroblasts were transduced with retroviruses encoding each of the indicated constructs and selected with puromycin. (**c**) Stable cell lines were lysed and proteins were resolved by SDS-PAGE. Immunoblot analyses using FLAG-specific antibodies were performed to evaluate expression of WT RTA and CS-RTAs. Detection of β-actin serves as a loading control. (**d**) Stable cell lines were fixed and stained with FLAG-specific antibodies. Protein expression and localization were visualized by indirect immunofluorescence microscopy. DNA was visualized by staining with DAPI.

Because manipulating CPB and CpG content can alter the translation efficiency of proteins^9^, we first confirmed that CS-RTA constructs were translated and functional for viral gene transactivation upon transient transfection of 293 T cells. All CS-RTA constructs efficiently transactivated promoters for ORF57 and ORF72, RTA-responsive MHV68 promoters, in comparison to WT RTA in luciferase reporter assays (Fig. 1a and b). As this is a steady-state assay and variations between WT and CS-RTA induction of the two different promoter constructs are not consistent, we reason that differences in promoter transactivation observed are likely the result of small variations in transfection efficiency for the individual samples. For viral complementation experiments, we generated stable NIH 3T12 fibroblast lines by transduction with retroviruses encoding WT RTA, CS-RTA1-5, or empty vector control. Immunoblot and indirect immunofluorescence analyses confirmed that WT RTA and CS-RTA proteins were expressed and localized to the nucleus in stable cell lines generated for complementation (Fig. 1c and d). The expression levels of CS-RTA constructs in immunoblot analyses was highest in the CS-RTA2 and CS-RTA4 stable cell lines, with CS-RTA4 most closely resembling WT RTA (Fig. 1c). For CS-RTA2, the enhanced CBP score may promote more efficient translation, while CS-RTA4 remains similar to WT RTA despite the nucleotide changes present (See Table 1).

**Figure 2:**
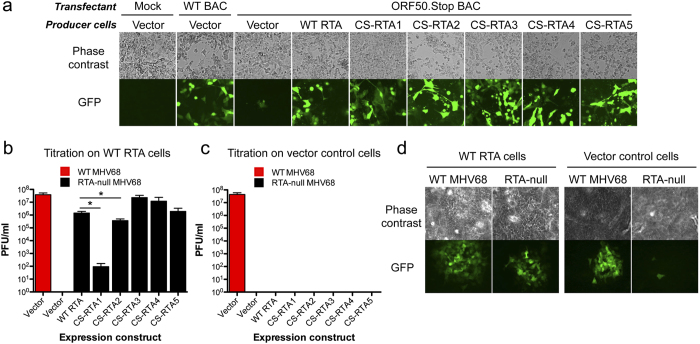
CS-RTAs complement RTA-null MHV68 replication. (**a**) Vector control, WT RTA, or CS-RTA stable cell lines were transfected as indicated with either WT MHV68 BAC or ORF50.stop MHV68 BAC. Phase contrast and epifluorescence microscopy to detect virus-encoded GFP were performed 8 days post-transfection to visualize cytopathic effect and viral spread within cultures. (**b** and **c**) Vector control, WT RTA, or CS-RTA stable cell lines were transfected with either WT MHV68 or ORF50.STOP MHV68 BAC, and viral stocks were produced. Viral titers for each stock were determined by plaque assay on either WT RTA (**b**) or vector control (**c**) stable cell lines. Results are means of triplicate samples. Error bars represent standard deviations. (**d**) Representative phase contrast and epifluorescence microscopic images demonstrating sporadic GFP-positive cells (see lower right panel) indicative of non-spreading infection by RTA-null virus produced in CS-RTA cells. Asterisks denote p < 0.05 as determined by student t-test.

To determine whether CS-RTA constructs support viral replication, vector control, WT RTA or CS-RTA-expressing cells were transfected with RTA-null ORF50. Stop MHV68 BAC^13^, and cells were observed over time for evidence of viral replication. As a positive control, vector control cells were transfected with WT MHV68 BAC. Three days post-transfection, GFP (expressed from the MHV68 BAC) was readily detectable in scattered individual cells, indicating that the cells were successfully transfected with ORF50.Stop BAC. In WT and CS-RTA-expressing cells, GFP fluorescence intensity increased and spread to neighboring cells in a manner analogous to WT MHV68 control (Fig. 2a). In contrast, GFP fluorescence remained dim and restricted to isolated cells in vector control cells transfected with ORF50.Stop BAC (Fig. 2a). Thus, complementation of the ORF50.Stop BAC by CS-RTA constructs, like WT RTA, enables propagation and cell-to-cell spread of RTA-deficient MHV68.

RTA-null MHV68 stocks from WT or CS-RTA complementation were generated by propagating two additional passages on cognate cell lines and titrated by plaque assay on WT RTA-expressing 3T12 fibroblasts. Titers of 10^5^ to 10^7^ PFU per ml were quantified for WT RTA and 4 of 5 CS-RTA stable cell lines (Fig. 2b). Interestingly, CS-RTA1, which had the most nucleotide changes and lowest CPB score of all CS constructs, was much less efficient than CS-RTA2-5, yielding titers of approximately 100 PFU per ml. However, CS-RTA2, which has an optimal CPB score and expressed better than WT RTA (Table 1 and Fig. 1c), also was significantly impaired for complementation (Fig. 2b). This suggests that factors beyond simple amino acid sequence impact the capacity for complementation. Plaques were not detected for any RTA-null MHV68 stocks when parallel plaque assays were performed on vector control cells (Fig. 2c), though single GFP + cells indicative of entry and genome delivery were identified by fluorescence microscopy (Fig. 2d). These data demonstrate that CS-RTA constructs complement RTA-null MHV68 replication without recombination that would enable growth on vector control cells. In addition, CPB and/or CpG content, which likely influence the efficiency of translation (see Fig. 1c), are important considerations for CS complementation constructs.

### Evaluating reversion during complementation

We further examined whether production of RTA-null MHV68 in CS-RTA expressing cells prevented WT reversion, as compared to production in WT RTA cells. Given the low yield, virus produced in CS-RTA1 cells was not included in downstream analyses. Virus stocks were concentrated by centrifugation to ca. 1 × 10^8^ PFU per ml in order to increase the sensitivity of detecting revertant WT viruses. Plaque assays were performed for concentrated RTA-null stocks on vector control 3T12 cell lines to identify stocks containing WT MHV68 capable of producing plaques in the absence of the helper gene. While virus produced on WT RTA expressing cells yielded plaques on control cells (reversion frequency in initial experiments of 1.0 PFU per 10^8^ complemented PFU), no plaques were detected for any virus stock derived from cells stably expressing CS-RTA constructs, although titers were comparable on WT RTA-expressing cells (Table 2). Of note, the reversion frequencies in our experiments using retroviral transduction to express RTA were lower than previous studies that used plasmid-based complementation^13^. While an explanation for this difference is not immediately evident, the previous study used a traditional plasmid-based approach, rather than the retroviral transduction method used in our study, to generate producer cells. We speculate that there likely were more copies of the helper gene present in producer cell lines in the previous study that effectively increased the potential for reversion through homologous recombination.

**Figure 3:**
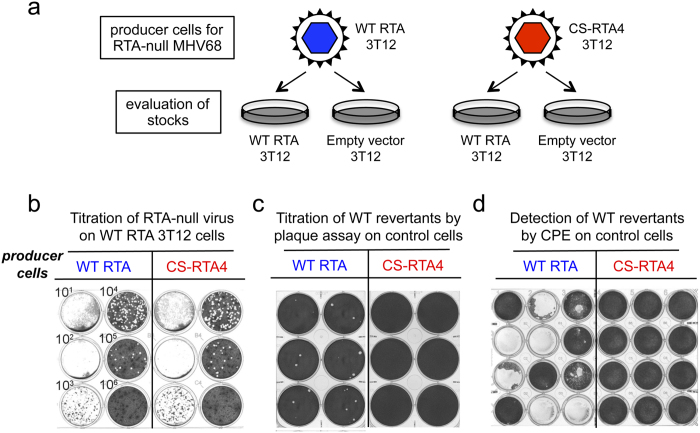
CS-RTA prevents WT reversion during complementation of RTA-null MHV68. (**a**) Schematic representation of approach to evaluate WT reversion in RTA-null virus stocks. (**b**) Plaque assay on WT RTA expressing cell line demonstrating approximately equivalent complemented viral titers for RTA-null virus stocks produced in either WT RTA or CS-RTA4 cells. (**c**) RTA-null MHV68 produced in either WT RTA or CS-RTA4 stable cell lines and concentrated to greater than 10^8^ PFU/ml was plated directly onto vector control cells in a plaque assay to test for the presence of WT revertant virus in stocks. (**d**) Undiluted RTA-null virus stocks were plated onto vector control 3T12s and observed for cytopathic effect. This assay is approximately 10-fold more sensitive in detecting replication-competent virus than standard plaque assays. A summary of reversion frequencies in all experiments is provided in Tables 2 and 3.

To further evaluate complementation by codon shuffling, we repeated the complementation experiment with WT RTA and CS-RTA4, the construct with the fewest number of nucleotide changes (109 nt, see Table 1 and Supplementary Fig. 1) from WT coding sequence, all present in the first 384 nucleotides of the CS construct. The strategy for evaluating WT reversion is highlighted in Fig. 3a. Each virus stock had comparable titers on WT RTA-3T12 cells (Fig. 3b), yet the virus produced in CS-RTA4 cells did not generate any plaques when plated on 3T12 cells without RTA complementation. In contrast, WT RTA-complemented viruses formed plaques on vector control 3T12 cells (Fig. 3c), with a reversion frequency of 1.12 PFU per 10^6^ complemented PFU. As a more sensitive test, we also performed cytopathic effect assays (CPE) to detect virus reversion. The MHV68 CPE assay is ca. 10-fold more sensitive than plaque assays for revealing the presence of replication-competent MHV68^14^. Virus stocks produced in WT RTA-expressing 3T12s induced CPE in 8 of 12 wells of control 3T12s, while stocks derived from CS-RTA4 cells did not yield CPE in any wells (Fig. 3d).

Finally, to test the reproducibility of these observations, we performed ten additional independent complementation trials with WT RTA and CS-RTA4 stable 3T12 cells (Table 3, repeats 2 to 11). Of the eleven virus stocks produced by complementation with WT RTA, eight exhibited reversion at a level of detection of ca. 1 WT virus PFU in 10^8^ complemented PFU, with reversion frequencies ranging from 0.12–11.98 per 10^6^ complemented PFU. In contrast, no revertant WT viruses were detected in any of the eleven virus stocks produced in CS-RTA4 stable cells. These results demonstrate that synthetic complementation vectors designed by codon shuffling effectively eliminate the risk of WT reversion when producing high-titer stocks of a replication-defective herpesvirus. Together, these data strongly suggest that codon shuffling has the capacity to limit WT reversion during complementation of mutant viruses. Moreover, based on data for CS-RTA4, we can reasonably predict that alteration of roughly 1/3^rd^ of nucleotides within a helper gene in a region extending approximately 150nt from a distinct lesion in a mutant herpesvirus is sufficient to eliminate reversion using this approach.

### Safety concerns of wild-type reversion in a model vaccine stock

A potential goal of herpesvirus vaccines is the preventive or prophylactic vaccination of individuals with increased risk factors for developing severe manifestations of infection. This could include immune suppressed persons, for whom it may be catastrophic to receive a vaccine in which WT virus is present, even at miniscule levels. While subunit vaccines against herpesviruses have been tested^15^,^16^,^17^,^18^, there currently is not a virus-like particle (VLP) equivalent vaccine formulation in trials. If available, such a vaccine could potentially provide increased protection due to the presence of a broader repertoire of viral epitopes against which to mount an immune response. Replication and latency-defective viruses offer a potential VLP platform, as these viruses essentially would be biologically inert upon inoculation into a recipient host. However, production of such vaccines would necessitate complementation via a producer cell line, and it would be necessary to ensure that such a vaccine was devoid of WT revertants.

**Figure 4:**
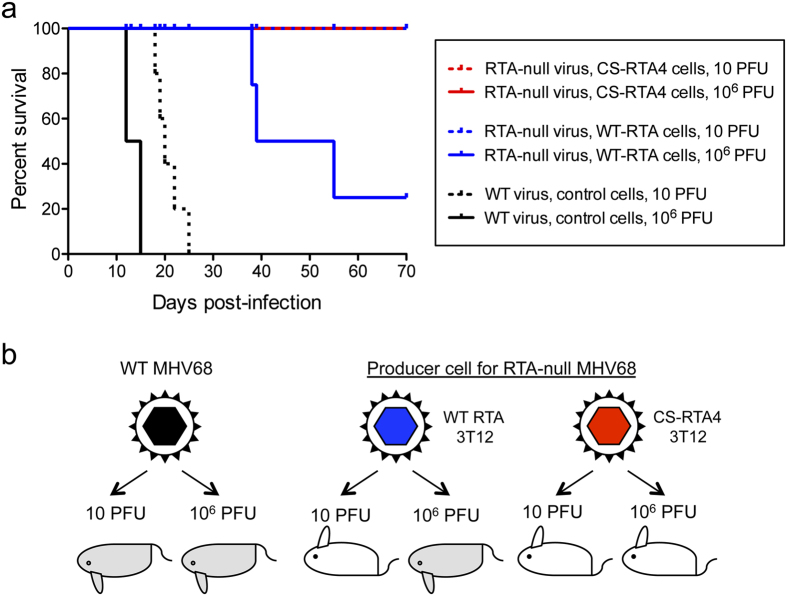
CS-RTA produced RTA-null virus is non-lethal in SCID mice. (**a**) SCID mice were inoculated with either high-dose (10^6^ PFU) or low-dose (10 PFU) of either WT MHV68, RTA-null MHV68 produced on WT-RTA stable cells with a reversion frequency of ca. 1 in 10^6^ PFU, or RTA-null MHV68 produced on CS-RTA4 stable cells with no detectable revertants. Mortality was monitored over time after infection. Differences in mortality following infection with 10 PFU or 10^6^ PFU of WT MHV68 were statistically significant in comparison to all other groups by log-rank Mantel-Cox test. (**b**) Schematic summary of results.

Infection of SCID mice with WT MHV68 causes lethality in 100% of infections^19^,^20^. To rigorously validate that RTA-null virus stocks derived by codon shuffling were devoid of replication-competent MHV68, we infected severe-combined immunodeficient (SCID) mice with RTA-null virus produced in WT RTA or CS-RTA4 cells. Infection with either 10 PFU or 10^6^ PFU of WT MHV68 served as positive controls for disease in these experiments. Mice infected with 10^6^ PFU of WT MHV68 succumbed by 12–15 dpi, while those infected with 10 PFU succumbed between 18–25 dpi (Fig. 4). Infection with 10^6^ PFU of the RTA-null MHV68 produced in WT RTA cells with a reversion rate of ca. 10 WT PFU in 10^6^ complemented PFU caused death in 3 of 5 animals between 38 and 55 dpi. However, no mortality occurred over a 70 day period for SCID mice infected with 10 PFU or 10^6^ PFU of RTA-null virus produced in CS-RTA4 cells. These data further confirm that application of codon-shuffling technology is suitable for generating high-titer, yet replication-defective, virus stocks. Moreover, this approach also allays the potential safety concerns of WT revertants in vaccine stocks.

## Summary and Conclusions

The propensity of herpesvirus genomes to undergo homologous recombination is a barrier to producing mutant virus stocks that require complementation with a helper gene provided *in trans*. We recoded a helper gene for *ORF50* which encodes the essential rhadinovirus lytic transactivator protein RTA using different permutations of our shuffling algorithm to maintain the amino acid sequence and codon usage, yet explore the impact of CPB and CpG content on complementation. The CS-RTA4 helper gene had ~100 nucleotides changed in the 5′ region of the construct that corresponded to the lesion present in the RTA-null mutant virus. The CPB of CS-RTA4 has an optimal score, and the construct expressed at levels comparable to WT RTA in the producer cell line. Importantly, the RTA-null virus grew to high titer in the CS-RTA4 producer line, but there was no evidence of reversion and replication of these stocks in non-complementing cells. To demonstrate safety, the infection of immune-deficient SCID mice with 10^6^ PFU of RTA-null virus caused no mortality, in marked contrast to 60% mortality caused by an RTA-null virus stock grown on the WT RTA producer cell line that generated revertants.

The application of this technology to produce replication-defective virus stocks will facilitate studies of early infection events in cell culture studies or pathogenesis in animal models, as codon shuffling obviates the caveat of contaminating WT viruses potentially influencing experimental results. We expect that this technique will be readily adaptable to other viral systems, such as adenovirus or poxvirus, in which reversion via recombination of a mutant viral genome with a helper gene frequently leads to WT virus contamination of mutant-virus stocks. In addition, codon shuffling provides an effective method for producing high-titer attenuated – or even replication-defective – mutant viruses that offer an avenue as ‘smart-design’ herpesvirus vaccines. Thus, we propose that replication-defective virus stocks produced with this technology will be safe to use in immunocompromised hosts.

## Methods

### Design of codon-shuffled RTA sequences

Codon bias is a phenomenon based on codon usage and codon occupancy in ribosomes. Codon bias influences codon-pair bias, which describes the frequency of paired codon occurrence in a given gene relative to a known data set^9^. Based on the experimentally determined codon usage for a certain species, there are expected frequencies with which distinct codon pairs should occur in a coding sequence. If codon pairs in a gene are overrepresented compared to the expected frequency, the codon pair score will be positive. Underrepresented codon pairs will have a negative score. The codon usage of each RTA-encoding ORF50 construct was determined relative to the codon usage table for *Mus musculus*. The relative adaptation of each codon was used to calculate the codon adaptation index for the entire gene. Codon-pair bias scores were calculated based on the codon-pair bias scoring human reference table, since codon pair bias is highly conserved among mammals.

CS-RTA1 was designed based on the max scramble algorithm previously described^6^,^7^. Briefly the algorithm involved stimulated annealing and bipartite matching to optimize the number of nucleotide changes and minimize homology in ORF50 while using the same set of codons. CS-RTA2 was designed with a search algorithm to minimize homology using the same set of codons, but with the aim of achieving a more optimal codon pair bias score. CS-RTA3 is a fusion of the first 381 nt of CS-RTA1 with the remainder of the WT RTA sequence. CS-RTA4 was designed with a similar algorithm as for CS-RTA2, but with parameters to restore a more optimal codon pair bias score, and was only applied to the first 381 nt of ORF50. CS-RTA5 is a fusion of the first 384 nt of CS-RTA2 with the remainder of the WT RTA sequence. Regions of homology less than 6 nucleotides were not counted against the score.

Unique CS-RTA sequences were synthesized by Blue Heron Biotechnology (now OriGene) with the addition of an N-terminal FLAG-tag. An internal *Bgl*II site in ORF50 was mutated in CS constructs to facilitate cloning. The source of ORF50 encoding RTA, plasmid psg50^13^, was found to have two mutations compared to the published reference genome (U97553.2^21^). The nonsynonymous C to T mutation at nucleotide 242 of ORF50 was repaired back to WT sequence. A second silent C to T mutation at nucleotide 1225 was left in the WT ORF50 sequence and was also present in CS-RTA3, CS-RTA4, and CS-RTA5. WT RTA was cloned into the *Xho*I site of pMSCV-puro (Clontech). CS-RTA1 and CS-RTA2 were cloned into the *Bgl*II and *Eco*RI sites of pMSCV-puro (Clontech). CS-RTA3, CS-RTA4, and CS-RTA5 were generated by splice-overlap extension PCR. CS-RTA3 and CS-RTA5 were cloned into the *Bgl*II and *Eco*RI sites of pMSCV-puro. CS-RTA4 was cloned into the *Bgl*II and *Xho*I sites of pMSCV-puro. Fidelity of cloning was verified by automated sequencing. Sequence alignments were performed using Geneious software.

### Cells and viruses

NIH 3T12 fibroblasts and BOSC23 ecotropic retroviral packaging cells were purchased from ATCC. Cells were cultured in Dulbecco’s modified Eagle medium (DMEM) supplemented with 10% fetal bovine serum, 2 mM L-glutamine, 100 units/ml penicillin, and 100 μg/ml streptomycin. Cells were cultured at 37 C in atmosphere containing 5% CO_2_. Murine stem cell virus (MSCV)-based retroviral vectors were produced by transfecting BOSC23 cells with empty pMSCV or individual pMSCV-RTA constructs using lipofectamine (Invitrogen) according to the manufacturer’s instruction. Two days post-transfection, retroviral supernatants were harvested and filtered through 0.45 μm filters (Merck Millipore) to remove cell debris. Filtered retroviruses were added directly to NIH 3T12 fibroblasts in culture medium supplemented with 4 μg/ml polybrene. Transduced cells were selected by adding 5 μg/ml puromycin two days post-transduction and expanded in the presence of puromycin for two weeks until puromycin resistant cells were obtained.

Wild-type MHV68^22^ or ORF50.Stop^13^ MHV68 BACs were transfected into either vector control cells or cell lines encoding either WT RTA or CS-RTA constructs 1–5 using lipofectamine and PLUS reagent (Invitrogen). Viral supernatants were harvested from transfected cell lysates seven days post-transfection and passaged two additional times on the appropriate cognate cell line to produce working stocks for experimentation. All viral stocks were harvested by two freeze-thaw cycles followed by centrifugation at 500 g for 10 min at 4 °C to remove cell debris. Viral stocks were concentrated by centrifugation at 35,000 g for 90 min at 4 °C followed by resuspension of virion pellets in fresh medium of 1/10 original volume.

### MHV68 plaque assay and cytopathic effect assay

Viruses were serially diluted and titrated by plaque assay as described previously^23^ on vector control 3T12 cells to evaluate reversion for RTA-null viruses and WT RTA 3T12 cells to determine titers of complemented RTA-null stocks. WT reversion titrations were performed by plating undiluted virus directly onto vector control cells in plaque assays on 6-well plates. Cells were fixed with formalin and stained with crystal violet seven days post-infection for plaque visualization and enumeration. Cytopathic effect assays were performed by incubating 50 μl of concentrated virus stocks with vector control 3T12 cells in 24-well plates. Cells were fixed and stained with crystal violet in formalin ten days post-infection, and cytopathic effect was observed.

### Mice and infections

CB.17 severe-combined immunodeficient (SCID) mice were purchased from Harlan laboratories (Envigo, Indianapolis, IN). All experiments were performed in accordance with a protocol approved by the Institutional Animal Care and Use Committee of Stony Brook University. 6 week old female CB.17 SCID mice were infected with 10 PFU or 10^6^ PFU of recombinant MHV68 in 0.5 ml cMEM by intraperitoneal injection.

### Statistical analyses

Statistical analyses were performed using GraphPad Prism software. Significant differences in plaque assays were defined using a two-tailed, paired student t-test in which CS-RTA complementation was compared to WT RTA. Significant differences in mortality of SCID mice were defined using a log-rank Mantel-Cox test wherein each infection group was compared individually to the other groups.

## Additional Information

**How to cite this article:** Li, G. *et al*. A codon-shuffling method to prevent reversion during production of replication-defective herpesvirus stocks: Implications for herpesvirus vaccines. *Sci. Rep.* **7**, 44404; doi: 10.1038/srep44404 (2017).

**Publisher's note:** Springer Nature remains neutral with regard to jurisdictional claims in published maps and institutional affiliations.

## Supplementary Material

Supplementary Figure

## Figures and Tables

**Table 1:** Parametric analyses of codon-shuffled constructs relative to wild-type RTA.

Construct	nt changes	Codon adaptation index	Codon-pair bias score	Codon-pair bias score per codon	CpG observed vs. expected ratio
WT RTA	0	0.7319	0.1846	0.0003	0.4524
CS-RTA1	642	0.7321	−67.6708	−0.1160	0.7489
CS-RTA2	614	0.7321	69.2674	0.1188	0.4411
CS-RTA3	136	0.7316	−24.1314	−0.0414	0.5670
CS-RTA4	109	0.7319	−6.8171	−0.0117	0.5244
CS-RTA5	133	0.7207	12.2735	0.0211	0.5262

**Table 2:** Reversion frequencies of RTA-null MHV68 produced in WT RTA or CS-RTA stable cell lines.

Helper gene used for complementation	Titer on WT RTA cells (PFU per ml)	Titer on control cells (PFU per ml)	Reversion per million viruses
WT RTA	11 × 10^8^	11	0.01
CS-RTA2	0.9 × 10^8^	Not detected*	Not detected
CS-RTA3	3.8 × 10^8^	Not detected	Not detected
CS-RTA4	6.0 × 10^8^	Not detected	Not detected
CS-RTA5	7.0 × 10^8^	Not detected	Not detected

*Below detection limit of 5 PFU per ml. P2 virus stocks (30 ml) were concentrated ca. 100 times by centrifugation. CS-RTA1 complemented virus samples were not included due to low virus titer in viral stocks (below 10^2^ PFU/ml).

**Table 3:** Reversion frequencies for WT RTA and CS-RTA4-complemented RTA-null virus stocks.

RTA-null MHV68 stocks produced in WT RTA stable 3T12 cells			
Repeat number	Titer on WT RTA cells (PFU per ml)	Titer on control cells (PFU per ml)	Reversion per million viruses
1	1.7 × 10^7^	19	1.12
2	5.4 × 10^7^	30	0.56
3	5.4 × 10^7^	Not detected*	Not detected
4	6.9 × 10^7^	8	0.12
5	6.0 × 10^7^	15	0.24
6	8.4 × 10^7^	690	8.21
7	4.2 × 10^7^	Not detected	Not detected
8	6.3 × 10^7^	15	0.24
9	4.2 × 10^7^	503	11.98
10	6.0 × 10^7^	38	0.63
11	3.9 × 10^7^	Not detected	Not detected
**RTA-null MHV68 stocks produced in CS-RTA4 stable 3T12 cells**			
**Repeat number**	**Titer on WT RTA cells (per ml)**	**Titer on control cells (per ml)**	**Reversion per million viruses**
1	2.0 × 10^7^	Not detected	Not detected
2	3.3 × 10^7^	Not detected	Not detected
3	3.0 × 10^7^	Not detected	Not detected
4	3.0 × 10^7^	Not detected	Not detected
5	3.6 × 10^7^	Not detected	Not detected
6	2.5 × 10^7^	Not detected	Not detected
7	2.7 × 10^7^	Not detected	Not detected
8	3.3 × 10^7^	Not detected	Not detected
9	2.9 × 10^7^	Not detected	Not detected
10	3.6 × 10^7^	Not detected	Not detected
11	2.7 × 10^7^	Not detected	Not detected

*Below detection limit of 5 PFU per ml. Plaque assay images of repeat 1 samples are shown in Fig. 3.
