# Supplementary material for: A codon-shuffling method to prevent reversion during production of replication-defective herpesvirus stocks: Implications for herpesvirus vaccines
Source: Sci Rep. 2017 Mar 13;7:44404. doi: 10.1038/srep44404 (PMC5347388; doi:10.1038/srep44404)
Supplement: Supplementary Figure [file srep44404-s1.pdf]

Supplementary Information

A codon-shuffling method to prevent reversion during production of replication-defective herpesvirus stocks: Implications for herpesvirus vaccines

Gang Li[1], Charles Ward[2#], Rukhsana Yeasmin[2#], Steven Skiena[2], Laurie T. Krug[3]*, and J. Craig Forrest[1*&]

[1]Department of Microbiology and Immunology and Center for Microbial Pathogenesis and Host Inflammatory Responses, University of Arkansas for Medical Sciences, Little Rock, Arkansas, USA

[2] Department of Computer Science and [3]Department of Molecular Genetics and Microbiology, Stony Brook University, Stony Brook, New York, USA

# Present addresses:

Charles Ward, Google,1600 Amphitheatre Pkwy., Mountain View, CA 94043

Rukhsana Yeasmin, Apple, 1 Infinite Loop, Cupertino, CA 95014

* Co-senior authors

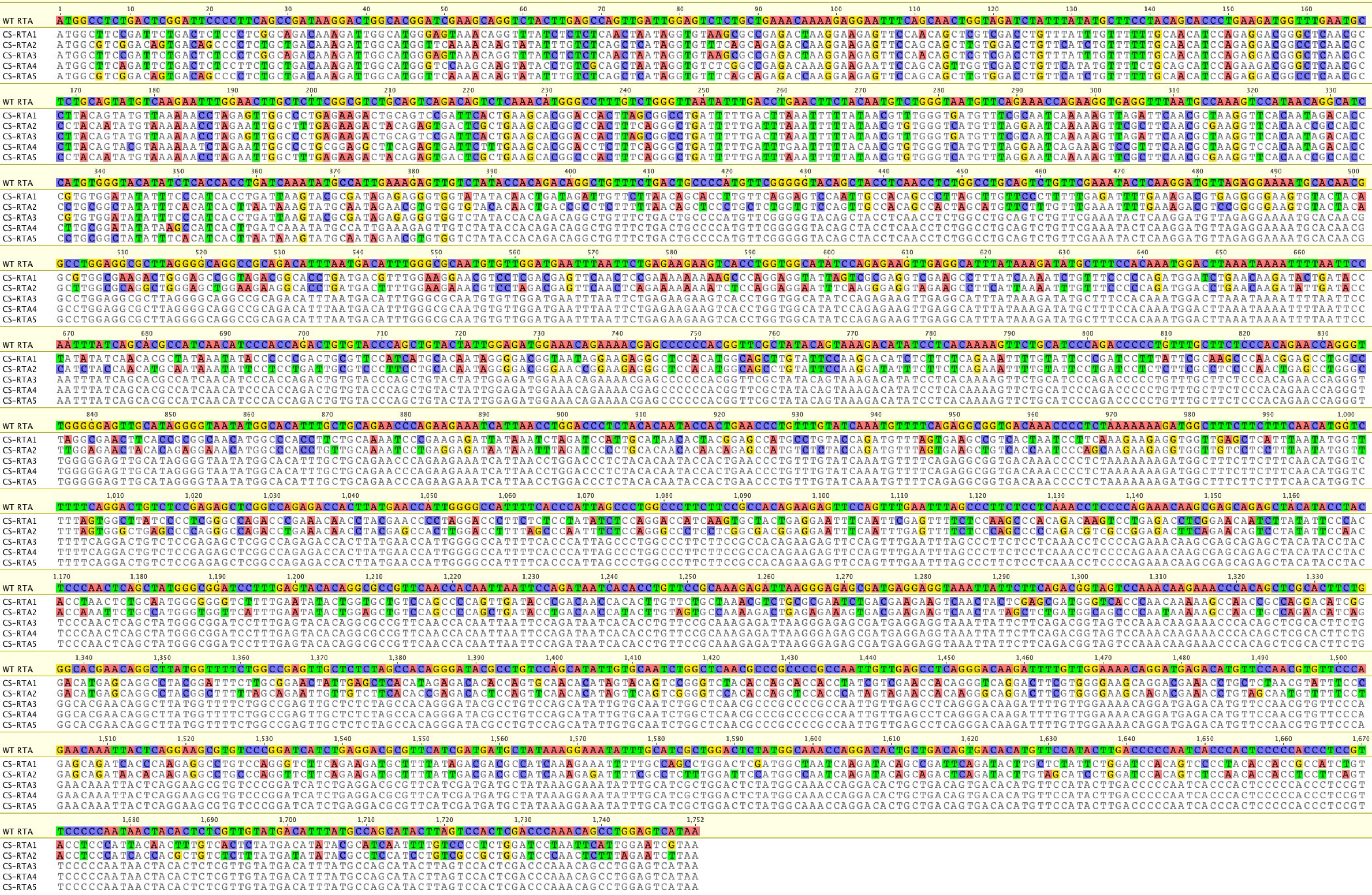

Supplementary Figure 1. Nucleotide alignments of codon-shuffled ORF50 sequences with WT MHV68 ORF50. Synthetic RTA-encoding ORF50 nucleotide sequences were derived using computer algorithms that take into account codon adaptation index and codon pair bias scores. Absolute codon content is preserved in the codon shuffling approach, while nucleotide sequences and thus nucleotide homology are disrupted. Shown are alignments of synthetic CS-RTA nucleotide sequences produced for this study with WT MHV68 ORF50.
